# Goal-directedness deficit in Huntington’s disease

**DOI:** 10.3758/s13415-025-01313-0

**Published:** 2025-06-02

**Authors:** Lee-Anne Morris, Sanjay Manohar, Kyla-Louise Horne, Laura Paermentier, Christina M. Buchanan, Michael J. MacAskill, Daniel J. Myall, Masud Husain, Richard Roxburgh, Tim J. Anderson, Campbell J. Le Heron

**Affiliations:** 1 Department of Medicine, University of Otago, Christchurch, New Zealand; 2 New Zealand Brain Research Institute, Christchurch, New Zealand; 3 Department of Experimental Psychology, University of Oxford, Oxford, UK; 4 Nuffield Department of Clinical Neurosciences, University of Oxford, Oxford, UK; 5 Department of Neurology, Auckland city hospital, Te Whatu Ora Health New Zealand, Auckland, New Zealand; 6 Centre for Brain Research Neurogenetics Research Clinic, University of Auckland, Auckland, New Zealand; 7 Department of Neurology, Christchurch hospital, Te Whatu Ora Health New Zealand, Christchurch, New Zealand

**Keywords:** Huntington’s disease, Apathy, Impulsive behaviour, Dimensions, Goal-directedness, Five-factor model

## Abstract

Apathy and impulsive behaviour co-occur in Huntington’s disease (HD), but these debilitating behavioural syndromes are multidimensional constructs, raising the question of which specific dimensions drive this relationship and the stability of the co-occurring dimensions across time. People with HD and controls completed multidimensional apathy and impulsive behaviour scales at baseline and 1-year follow-up. A principal component analysis was performed on pooled data (*n* = 109) to identify components and factor loadings of subscales. Linear mixed models were used to examine differences in components between groups and timepoints. Three meaningful components emerged. Component 1 comprised positive loading for dimensions of apathy and impulsive behaviour pertaining to goal-directedness, namely attention, planning, initiation, and perseverance. In contrast, other dimensions of apathy and impulsive behaviour loaded onto components two and three in opposite directions. People with HD only scored worse than controls on the goal-directedness component. All components remained stable over time and closely resembled factors from the five-factor personality model. Component 1 mapped onto the factor conscientiousness, component 2 to extraversion, and component 3 to neuroticism. The clinical overlap between apathy and impulsive behaviour in HD relates to goal-directedness, whilst other dimensions of these constructs did not overlap.

## Introduction

Huntington’s disease (HD) is an autosomal dominant neurodegenerative disease caused by *CAG* repeat expansion in the huntingtin gene, with mutant huntingtin expression. Early pathological changes are evident in the caudate nucleus in vivo, with loss of GABAergic medium spiny neurons in the striatum a hallmark of HD. Widespread neurodegeneration is evident postmortem. Clinically, HD is characterised by a triad of motor impairments, including chorea, cognitive decline, and neuropsychiatric disturbances. In addition to increased irritability, perseverations, and depression, apathy is highly prevalent in HD (Martinez-Horta et al., 2016).

Apathy is generally considered a clinical construct that manifests as a syndrome with specific symptoms, although people can experience a range of motivation in normal health. Broadly, apathy is defined as a quantitative reduction in goal-directed behaviour compared with previous levels of functioning (Robert et al., 2018). A wealth of literature demonstrates an increase in both prevalence and severity of apathy over time, across all stages of Huntington’s disease (Connors et al., 2023; Ruiz-Idiago et al., 2023; Tabrizi et al., 2013; Thompson et al., 2012; van Duijn et al., 2014). As such, it has been suggested that apathy is intrinsic to the disease (Thompson et al., 2012). Apathy in HD is associated with changes in subcortical and cortical regions, including the striatum, amygdala, dorsal anterior cingulate cortex, and anterior insula (De Paepe et al., 2021; Delmaire et al., 2013; Martínez-Horta et al., 2018; van den Bogaard et al., 2011). Experimental tasks show behavioural differences as a function of apathy in HD, namely increased sensitivity to costs associated with obtaining rewarding outcomes, leading to a shift in the subjective valuation of what is worth working for (Morris et al., 2024a) and insensitivity to losses (McLauchlan et al., 2019). Carers report that patients do very little without prompting, whilst patients report that they just “can’t be bothered.”

Impulsive behaviour, in contrast to apathy, is not necessarily a clinical syndrome and can be a personality trait or a transient state that emerges in specific contexts. Impulsive behaviour is defined as rapid, but often premature actions, without appropriate foresight (Dalley & Robbins, 2017). The trajectory of impulsive behaviours in HD is not well understood. The handful of studies examining the neural regions associated with impulsive behaviours in HD point to overlapping brain regions with those associated with apathy (Gray et al., 2013; Morris et al., 2022; Rao et al., 2014).

Rather than occupying opposite ends of a behavioural axis, the co-occurrence of apathy and impulsive behaviour in Huntington’s disease, as well as other brain disorders, is increasingly recognized (Kok et al., 2021; Lansdall et al., 2017; Morris et al., 2024b; Petitet et al., 2021; Sinha et al., 2013; Torrente et al., 2011; Velligan et al., 2002; Zhao et al., 2016). Both apathy and impulsive behaviours can be viewed as disruptions to goal-directed behaviour. These two distinct symptomologies may arise when a common neural system underlying goal-directed behaviour is disrupted (Morris et al., 2022; Sinha et al., 2013). One inherent intricacy is that both apathy and impulsive behaviour are multidimensional constructs, each encompassing a range of behavioural changes. Thus, the co-occurrence of these two syndromes may be driven by associations between specific dimensions of each. Investigating this question may shed mechanistic light on whether and how these behaviours may be intrinsically linked. Furthermore, their relationships over time may reveal additional insights, in the setting of progressive neurodegenerative disease.

Expressly, apathy is conceptualised as having cognitive, behavioural, social, and emotional dimensions (Levy & Dubois, 2006; Marin et al., 1991), although there is ongoing debate about these domains (Dickson & Husain, 2022). The Apathy Motivation Index (AMI) (Ang et al., 2017) is comprised of social, behavioural, and emotional dimensions, whilst the Apathy Evaluation Scale (AES) (Marin et al., 1991) has behavioural, cognitive, and emotional dimensions. With regards to impulsive behaviours, motoric impulsivity includes premature responding, and reduced ability to inhibit actions, whilst decisional impulsivity includes increased risk-seeking behaviour, lack of appropriate forethought leading to rapid decisions, and preference for immediate rewards (Dalley & Robbins, 2017). The Barratt Impulsiveness Scale 11 (BIS-11) encompasses motor impulsiveness, attentional impulsiveness and nonplanning impulsiveness (second order factors) (Patton et al., 1995), whilst the UPPS-P Impulsive Behaviour Scale includes risk-seeking (sensation seeking), persistence and planning (lack of premeditation, lack of perseverance), and emotional reactivity (positive and negative urgency) subscales (Whiteside & Lynam, 2001). The UPPS-P’s five subscales can be understood within an influential and widely accepted model of personality—the five factor model, in which individual traits are rated along spectrums of extraversion, agreeableness, conscientiousness, neuroticism, and openness (McCrae & Costa, 1985). In summary, whilst apathy and impulsive behaviours are useful broad terms to describe behavioural change, they each encompass numerous dimensions. Understanding the disrupted processes giving rise to the co-occurrence of apathy and impulsive behaviours necessitates a deeper examination of these dimensions.

One problem when examining the co-occurrence of apathy and impulsive behaviour dimensions using a plethora of subscales is that subscales can be highly correlated within questionnaires, and within individuals (Petitet et al., 2021). Examining the relationships between all subscales would necessarily require multiple comparisons of highly correlated data. A solution is to pool subscales together and reduce the data dimensionality by using a principal component analysis (Jolliffe & Cadima, 2016). This takes the correlation between subscales into account, as well as identifying latent relationships in the data. By revealing which subscales load into shared components, and the strengths of these loadings, it is possible to determine which apathy and impulsive behaviour dimensions cluster together or co-occur. One previous study has used principal component analysis to examine co-occurring apathy and impulsivity, but total scores were used for the principal component analysis (PCA) (e.g., AES total, BIS-11 total), not subscale scores (Lansdall et al., 2017). Furthermore, their question of interest pertained to differences in patient-rated, self-rated, and behavioural-task derived measures of these behaviours, not the interrelationship of dimensions of them.

In the present study, we used principal component analysis to examine the relationships between apathy and impulsive behaviour subscales from the AES, AMI, BIS-11, and UPPS-P in people with Huntington’s disease and healthy controls at baseline and 1 year follow-up. In keeping with the idea that there are shared mechanisms underlying differing aspects of apathy and impulsive behaviours, we hypothesised that 1) the dimensions of apathy and impulsive behaviours would form distinct components that each encompassed contributions from both constructs; 2) people with HD would score higher than controls on a shared apathy-impulsive behaviour component related to goal-directed behaviour, and 3) this apathy-impulsive behaviour component would worsen by 1 year follow-up in people with Huntington’s disease but remain stable in healthy controls.

## Methods

### Ethics

The study was approved by the Health and Disability Ethics Committee of the New Zealand Ministry of Health (21/CEN/242). Written consent was obtained from all participants, and research was conducted in accordance with the Declaration of Helsinki.

### Participants

Participants were part of a larger Huntington’s disease study, reported elsewhere (Morris et al., 2024b). In brief, people with genetically confirmed expansion of the Huntingtin gene (premanifest to mild motor manifest disease) were recruited from two specialist HD clinics (Auckland and Christchurch, New Zealand, *n* = 42), and healthy controls were recruited from local databases. Of those, 19/20 (95%) controls and 28/42 (67%) people with Huntington’s disease were seen at 1 year follow-up. There were no significant baseline differences in age, motor disease severity, or apathy (AES score) between people with HD who did and those who did not attend the 1 year follow-up, whilst people who did not attend follow-up had on average lower MoCA scores (t_(15.2)_ = −2.1, *p* = 0.05). A summary of baseline demographic and behavioural data is presented in Table 1.

**Table 1:** Demographic and behavioural characteristics of participants

	Controls	HD	Test statistic
Participants	*n = 20*	*n* = 42	
Age	51 (18)	52 (14)	*t(30.6) =−0.2, p* = 0.8
Male: Female (%male)	8:12 (40%)	20:22 (48%)	*Χ*^*2*^*(*38, *n* = 62) = 37.4, *p* = 0.5
UHDRS - TMS /124	*NA*	13.8 (12.6)	
TFC /13	*NA*	11.3 (1.9)	
CAG repeat length	*NA*	42 (2)	
CAP score	*NA*	95 (20.6)	
MoCA /30	27.8 (1.9)	26.2 (4.7)	t(59.1) = 1.9, *p* = 0.07
SDMT (no ceiling)	52.8 (11.3)	38.4 (16)	t(44.9) = 4, ***p* = 0.0002**
**Apathy**			
AES-s /72	24.6 (4.4)	31.2 (9.6)	t(57.8) = −3.6, ***p* = 0.0006**
*Behavioural*	*6 (1)*	*8 (3)*	
*Cognitive*	*11 (2)*	*14 (5)*	
*Emotional*	*3 (1)*	*4 (1)*	
AMI /72	17.8 (6.5)	23.7 (9.2)	t(51.1) = −2.9, ***p* = 0.006**
*Behavioural*	*6 (3)*	*9 (5)*	
*Social*	*7 (4)*	*9 (5)*	
*Emotional*	*5 (3)*	*6 (4)*	
**Impulsivity**			
BIS-11 /120	53.5 (8.9)	60.7 (13.3)	t(41.9) = −2.2, ***p* = 0.03**
*Attentional*	*14 (3)*	*16 (4)*	
*Non-planning*	*20 (4)*	*23 (6)*	
*Motor*	*20 (3)*	*22 (4)*	
UPPS-P /236	115 (23.7)	124 (30)	t(47.2) = −1.3, *p* = 0.2
*Lack of perseverance*	*17 (5)*	*21 (6)*	
*Lack of premeditation*	*21 (6)*	*22 (5)*	
*Urgency (positive)*	*21 (8)*	*26 (11)*	
*Urgency (negative)*	*24 (8)*	*26 (9)*	
*Sensation seeking*	*32 (8)*	*30 (8)*	

Data are mean scores (standard deviations)

AES-s = Apathy Evaluation Scale (self-report); AMI = Apathy Motivation Index; BIS-II = Barratt Impulsiveness Scale – II; CAG = Cytosine adenine guanine; CAP = CAG age product; MoCA = Montreal Cognitive Assessment; TFC = Total functional capacity; UHDRS - TMS = Unified Huntington’s Disease Rating Scale Total Motor Score; UPPS-P = UPPS-P Impulsive Behaviour Scale

### Clinical and behavioural measures

Participants completed the self-report versions of two apathy scales (Apathy Evaluation Scale (AES), Apathy Motivation Index (AMI)) (Ang et al., 2017; Marin et al., 1991), and two impulsive behaviour scales (Barratt Impulsiveness Scale - 11 (BIS-11), UPPS-P impulsive behaviour scale) (Patton et al., 1995; Whiteside & Lynam, 2001). Although participants also had clinician-rated short Problem Behaviours Assessment (sPBA) apathy scores, we did not utilise these in the current study, as we were interested in the *dimensions* of apathy, which the sPBA does not provide, and their associations with dimensions of impulsive behaviour. Behavioural scales were administered in a quiet office by a trained researcher. Participants could choose to either read and complete questionnaires themselves or have questions read aloud to them one by one, with response options displayed in front of them to select. Cueing and prompting were provided as needed to best support participants with cognitive impairments. This included covering questions with a sheet of paper and only displaying the current question, giving extra time to answer questions, asking participants to verbalise answers to ensure they have understood the question and were responding appropriately and also encouraging participants to ask for clarification if they did not understand the question.

**Fig. 1:**
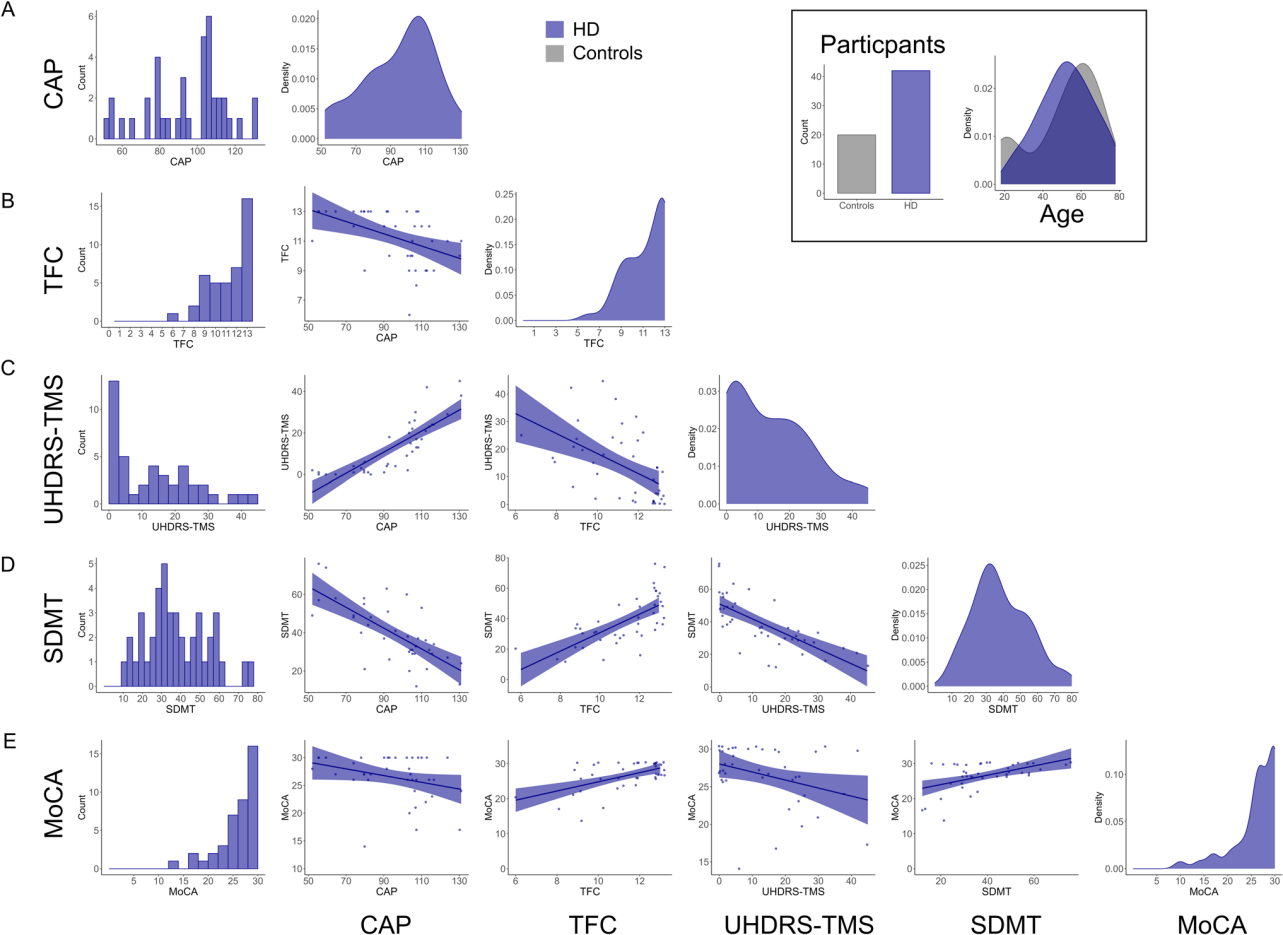
Baseline characterisation of the HD cohort. **A**) Distribution of CAP scores; **B**) Total functional capacity (TFC) scores; **C**) Unified Huntington’s disease rating scale – total motor scores (UHDRS-TMS); **D**) Symbol digit modality test (SDMT) scores; **E**) Montreal cognitive assessment (MoCA) scores. Inset shows number of control and HD participants and age distributions. To note, the relationships depicted in these plots are for visual display purposes only

Motor disease severity and functional capacity were measured by using the Unified Huntington’s Disease Rating Scale Total Motor score (UHDRS-TMS) and total functional capacity (UHDRS-TFC) section, respectively, by an experienced neurologist (TA, RR, or CLH) (Huntington Study Group, 1996). CAP score was calculated as per Warner et al. (2022) (CAP = age x (cag - 30)/6.49) (Fig. 1). Participants completed the same testing battery at 1 year follow-up.

### Statistical analysis

Differences between HD and controls were tested using Welch’s *t*-test or chi-squared tests, depending on the variables.

#### Principal components analysis for apathy and impulsive behaviour measures

We conducted a PCA to reduce the data dimensionality and explore the underlying data structure. Data from multiple questionnaires (AMI, AES, BIS-11, UPPS-P) was combined and each questionnaire’s subscales’ scores were used. Missing data were as follows: two people were missing AES scores; three were missing AMI scores; five were missing UPPS-P scores; and 13 BIS-11 scores. All participants had at least one measure of apathy and impulsive behaviour at baseline. No follow-up data were missing. Missing data were imputed by using *missMDA* in R, a method chosen given its applicability to multivariate data, iterative process, and relevance for PCA. For the BIS-11, we used second order factor subscales as described in Patton et al. (1995) (Attentional impulsivity = attention and cognitive instability; Motor impulsivity = motor and perseverance; Nonplanning impulsivity = self-control and cognitive complexity). Data from healthy controls and people with HD were pooled, as well as baseline and follow-up data (*n *= 109; baseline *n *= 62; follow-up *n = 47)*. The PCA identified the components of apathy and impulsive behaviour subscales that explained the most data variance. To determine the adequacy of the sample size, we used the Kaiser-Meyer-Olkin measure and Bartlett’s test of sphericity. Component loadings > 0.3 or < −0.3 were considered meaningful, as per recommendation (Field, 2013). The PCA (centred, scaled) was performed by using *FactoMineR* in R (R Core Team, 2024).

We fitted linear mixed effects models for each PCA-derived component separately to examine whether 1) component scores differed between people with HD and controls, 2) component scores changed over 1 year, and 3) change over time differed between people with HD and controls. Predictor variables group (HD/control), timepoint (baseline/follow-up) and their interactions were included, with component score as the outcome variable. To account for repeated measures per individual, data was nested within subjects by including subject as a random effect.

$$component\ score \sim group\times timepoint + (1 | subject)$$

To examine clinical and disease variables associated with apathy/impulsive behaviour components derived from PCA, linear mixed models were fitted to each component separately, with predictor variables of motor disease severity (UHDRS-TMS), cognition (SDMT), CAP score, and sex. Multicollinearity was assessed by using the variance inflation factor using the *car* package in R. The variance inflation factor for predictors of each model, respectively, were < 5 (no multicollinearity).

Lastly, we performed exploratory analyses, examining the relationship between component scores and sPBA-irritability and sPBA-perseverations.

## Results

### Demographic and clinical results

People with Huntington’s disease had significantly higher total scores on AES and AMI apathy scales and the BIS-11 impulsivity scale, whilst total scores on the UPPS impulsivity scale did not differ significantly compared to controls. In HD, linear regression demonstrated significant associations between sPBA apathy scores and AES and AMI apathy scores, respectively (AES: $\upbeta$ = 1.7, *t* = 5.2, *p* < 0.0001; AMI: $\upbeta$ = 0.9, *t* = 2.8, *p* = 0.008). There were no significant differences in age or sex between groups (Morris et al., 2024b) (Table 1).

### Principal component analysis

The sample size was adequate for analysis (Kaiser-Meyer-Olkin stat = 0.88) and correlations between items were sufficiently large for PCA (Barlett’s test of sphericity_91_ = 1,021, *p* < 0.001). The first three components were retained based on the Kaiser criterion (eigenvalues > 1), as well as examination of scree plot elbows. Together these explained 71% of the total variance in the data, an accepted cutoff (Jolliffe & Cadima, 2016). Component loadings are shown in Table 2. To determine whether the components themselves were meaningful, we examined the individual items of subscales comprising each component.

**Table 2:** Rotated component matrix extracted from principal component analysis

	Component 1	Component 2	Component 3
AES cognitive	**0.325325**	0.209607	0.072111
AES behavioural	**0.31894**	0.082369	−0.08642
AMI behavioural	**0.322922**	0.196708	0.031704
BIS-11 attentional	**0.339099**	−0.0996	−0.05038
BIS-11 non-planning	**0.322233**	−0.00251	−0.00219
UPPS-P lack of perseverance	**0.331128**	0.018291	0.101247
AES emotional	0.171234	**0.387511**	0.20972
AMI social	0.129306	**0.474118**	−0.0818
UPPS-P lack of premeditation	0.237068	**−0.30146**	0.212745
UPPS-P sensation seeking	0.021593	**−0.53557**	0.295136
AMI emotional	0.065783	0.087956	**0.799881**
UPPS negative urgency	0.294017	−0.14391	**−0.35926**
BIS-11 motor	0.280092	−0.28505	−0.02982
UPPS positive urgency	0.296243	−0.19484	−0.14396

Apathy (AES, AMI) and impulsivity (BIS-11, UPPS-P) subscale loadings onto the first three principal components, from principal component analysis. Factor loadings > 0.3 or < −0.3 were considered meaningful and are shown in bold

#### Component 1

Component 1 comprised positive loadings for the following subscales: AES behavioural (0.32), AMI behavioural (0.33), AES cognitive (0.33), BIS-11 attentional impulsivity (0.34), BIS-11 nonplanning impulsivity (0.32), and UPPS-P lack of perseverance (0.33). No negative loadings were evident. Despite the differing nomenclature of subscale names, some clear behavioural themes were apparent. Behaviour initiation was captured by the following subscale items: AES behavioural: *I get things done during the day/Someone has to tell me what to do each day*; AMI behavioural: *When I decide to do something, I am able to make an effort easily/I get things done when they need to be done* *without requiring reminders from others/I don’t like to laze around;* and AES cognitive: *Getting things done during the day is important to me/Getting things started on my own is important to me*. Perseverance was captured by items in the following subscales: UPPS lack of perseverance: *I generally like to see things through to the end/I finish what I start/I tend to give up easily*; AMI behavioural: *When I decide to do something, I am motivated to see it through to the end*; and AES cognitive: *Seeing a job through to the end is important to me*. Attention was captured by items in the following subscales: BIS-11 attentional: *I don’t pay attention/I concentrate easily*; UPPS lack of perseverance: *I concentrate easily.* Planning was captured as follows: BIS-11 nonplanning: *I save regularly/I plan tasks carefully/I plan trips well ahead of time*; and UPPS lack of perseverance: *Sometimes there are so many little things to be done that I just ignore them all/I am able to pace myself so as to get things done on time.*

**Fig. 2:**
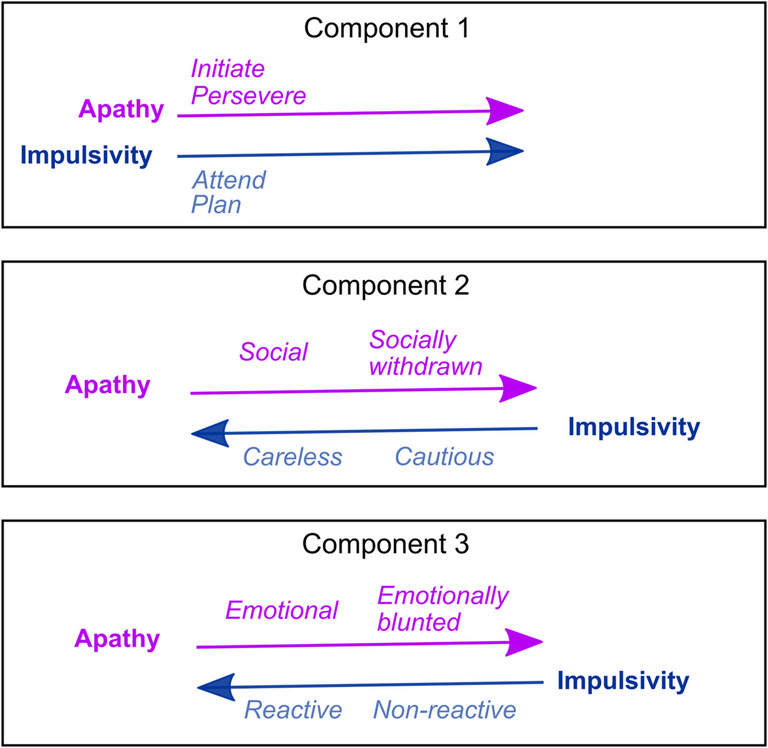
Components from apathy and impulsive behaviour subscales. Component 1 captured a core overlap between dimensions of apathy and impulsivity related to goal-directedness (attention, planning, initiation and perseverance), with loadings in the same direction. In contrast, components 2 and 3 did not demonstrate overlap between apathy and impulsivity dimensions. Rather, the various apathy and impulsivity dimensions loaded in opposite directions onto these components. Direction of arrows indicates worsening of the construct (more apathetic, more impulsive)

**Fig. 3:**
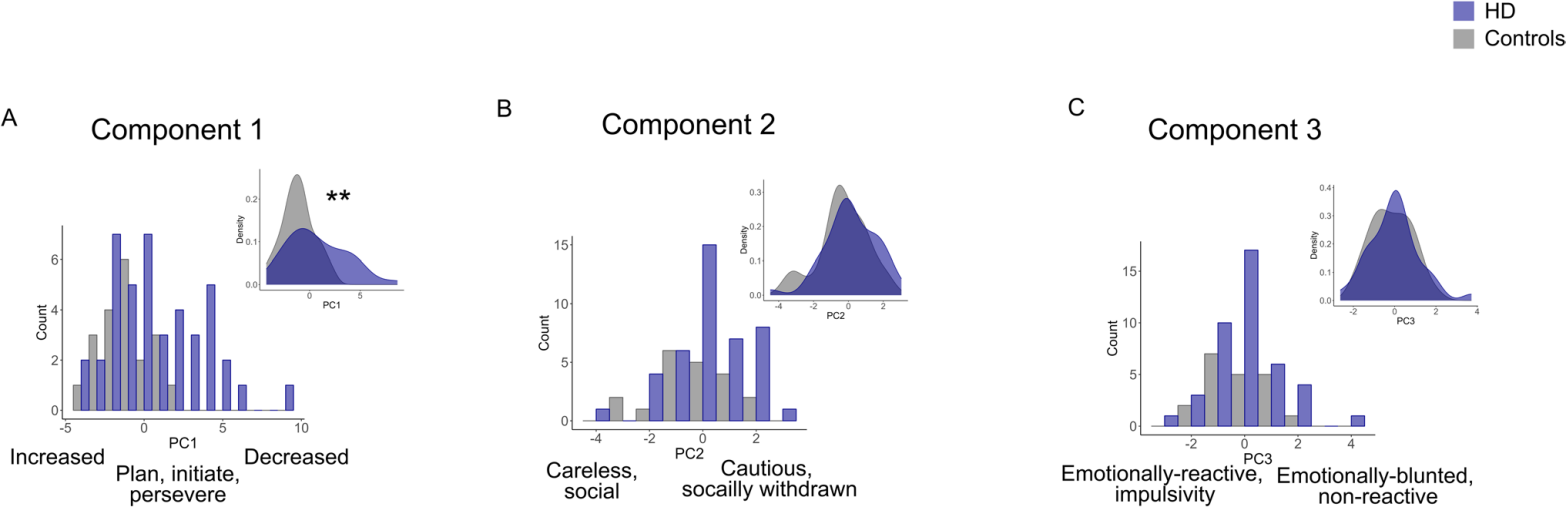
Individual scores on components. Component scores for people with Huntington’s disease (blue) and healthy controls (grey), collapsed across timepoints. **A**) Component 1 captured a core overlap between dimensions of apathy and impulsivity related to goal-directedness (attention, planning, initiation and perseverance) with higher scores indicating reduced (worse) goal-directedness. In contrast, components two (**B**) and three (**C**) did not demonstrate overlap between apathy and impulsivity dimensions, but rather, the various apathy and impulsivity dimensions loaded in opposite directions onto these components. **p < 0.01

Thus, this component described goal-directedness, encompassing planning, initiation, and perseverance of behaviours, as well as overall attention. Higher scores on this component described a goal-directedness deficit characterised by poor planning and attention, reduced behavioural initiation, and poor perseverance—an apathetic-impulsive phenotype (Fig. 2 & 3A).

#### Component 2

Component 2 comprised positive loadings for AMI social (0.47) and AES emotional (0.39) subscales. Negative loadings from UPPS-P lack of premeditation (−0.3) and UPPS-P sensation seeking (−0.54) were evident. The AMI social subscale has items, such as *I enjoy doing things with people I have just met; I go out with friends on a weekly basis; I start conversations without being prompted.* The AES emotional subscale has two items: *I approach life with intensity; when something good happens, I get excited.* The AES emotional apathy and AMI emotional apathy questions, although both named *emotional,*^1^ differ in terms of emotional valence. Whereas AES questions pertain to positive emotion, AMI emotional questions mostly pertain to negative emotion, which may explain the absence of the AMI emotional in this component (and lack of overlap in general, across all components). An increase in socially withdrawn behaviour, and emotional blunting to positively valenced stimuli increased scoring on this component. Lower scores on the UPPS-P lack of premeditation subscale indicate cautiousness (*I have a reserved and cautious attitude towards life/I am a cautious person*). The sensation-seeking subscale comprises items, such as *I would enjoy parachute jumping; I would enjoy the sensation of skiing very fast down a high mountain slope.* Such behaviour is not in reaction to something but is rather the goal-directed pursuit of an experience or *high* (positive emotion)*.* Higher sensation seeking decreased scores on this component.

Overall, higher scores on this component described a socially withdrawn phenotype, characterised by withdrawal from friends and not meeting new people or initiating social interactions. Furthermore, this was associated with cautiousness, rather than adrenaline-seeking behaviours or acting without thinking things through. Blunting to positive emotion also characterised high scorers on this component, which aligns well with the reduced pursuit of emotional highs/excitement (sensation seeking). In contrast, lower scores on this component described a social, thoughtless and adventure-seeking phenotype (Figs. 2 & 3B).

#### Component 3

Component 3 comprised one positive loading, AMI emotional apathy (0.8) and one negative loading, UPPS-P negative urgency (−0.36). The AMI emotional subscale has items such as, *I feel sad or upset when I hear bad news; I feel awful if I say something insensitive.* Higher scores (emotional blunting) led to an increase in this component score. Items on the UPPS-P negative urgency subscale include things like, *When I feel bad, I will do things I later regret to make myself feel better now; I have trouble controlling my impulses; when I am upset I act without thinking.* Specifically, this subscale pertains to rash reactions in response to negative emotions/stimuli. That this subscale has opposite loading direction to the AMI emotional subscale intuitively makes sense; emotional reactions (as captured by the negative urgency subscale) depend on sensitivity to emotion in the first place. Lower scores on this component described a nuanced emotionally reactive impulsivity, characterised by poorly thought-through behaviours in response to negative emotion or negatively valenced stimuli, associated with emotional sensitivity rather than blunting. Higher scores described the opposite—an emotionally blunted phenotype with reduced reactivity to negative emotional stimuli (Figs. 2 & 3C).

### Goal-directedness deficit in people with Huntington’s disease

**Fig. 4:**
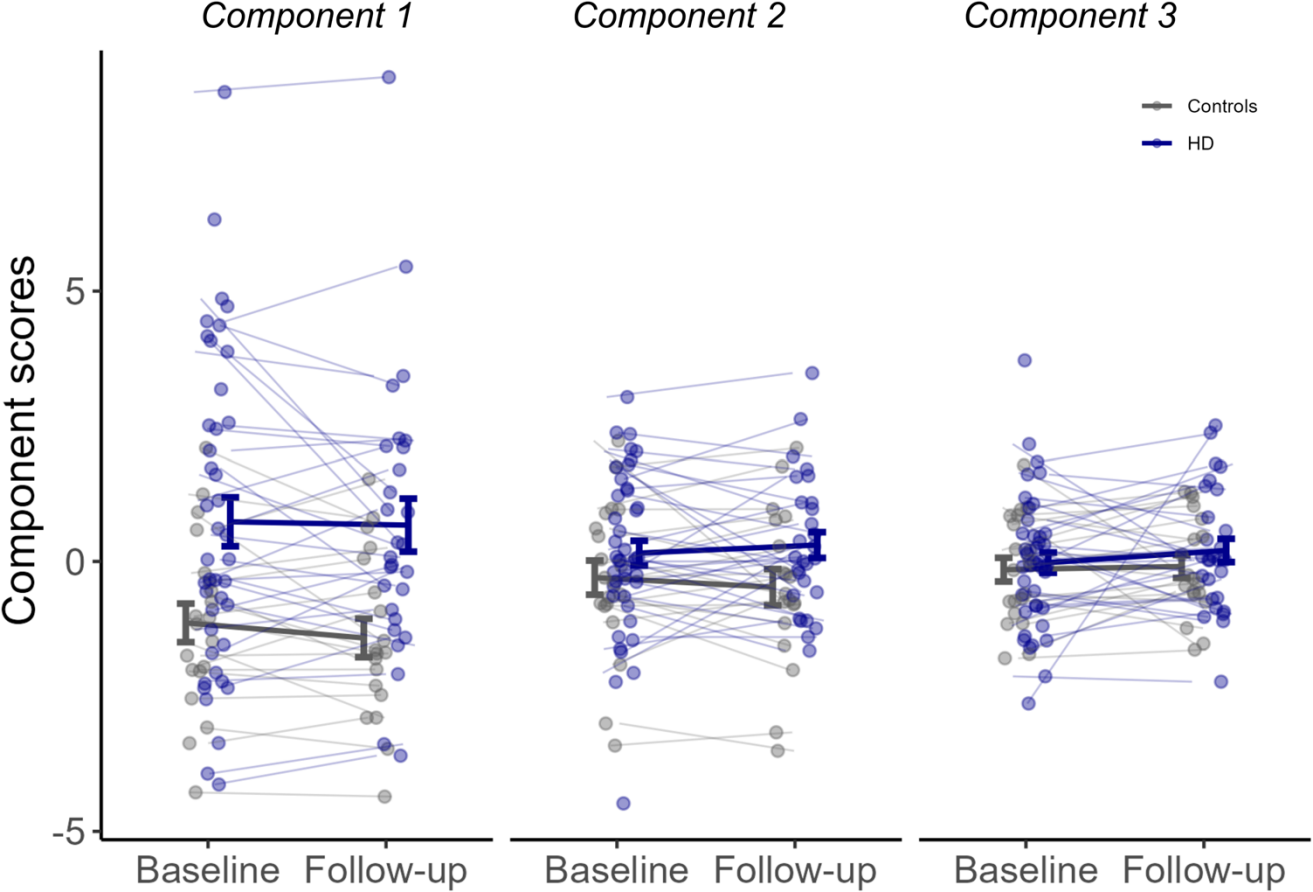
Individual component scores over 1 year. Individual scores for the first 3 principal components at baseline and 1 year follow-up, plotted for people with Huntington’s disease (blue) and healthy controls (grey). People with Huntington’s disease scored significantly higher (worse) on average on component 1 (Goal-directedness) compared to controls (*p* = 0.007) with no significant differences between groups on components 2 or 3. On average, components remained stable over time. Error bars show standard error of the mean

To determine whether components differed between groups cross sectionally or over time, or if group differences over time were evident, we fitted linear mixed models (see statistical analysis). There was evidence for a significant difference between groups on component 1 (goal-directedness), with people with HD scoring higher (worse) than controls (Group: $\beta$ = 1.9, *t* = 2.8, *p* = 0.007). In contrast, there was no evidence for significant differences between groups on components 2 or 3 (Component 2: Group: $\beta$ = 0.5, *t* = 1.7, *p* = 0.25; Component 3: Group: $\upbeta$ = 0.13, *t* = 0.4, *p* = 0.67) (Fig. 4).

For all components, there was no evidence for significant interactions between group and time (Component 1: Group $\times$ Time: $\upbeta$ = −0.02, *t* = −0.07, *p* = 0.95; Component 2: Group $\times$ Time: $\upbeta$ = 0.14, *t* = 0.45, *p* = 0.65; Component 3: Group $\times$ Time: group: $\upbeta$ = 0.18, *t* = 0.51, *p* = 0.61) and no evidence for significant main effects of time (Component 1: Time: $\upbeta$ = −0.3, *t* = −1.0, *p* = 0.33; Component 2: Time: $\upbeta$ = −0.13, *t* = −0.55, *p* = 0.59; Component 3: Time: $\beta$ = 0.04, *t* = 0.15, *p* = 0.88). Overall, component scores did not significantly differ from baseline to follow-up (Fig. 4).

### Disease severity and component scores in HD

To examine associations between cognitive and motor aspects of HD and component scores, linear mixed models were fitted to component scores (each component separately), with predictor variables motor disease severity (UHDRS-TMS), cognition (SDMT), CAP score and sex. Higher CAP score was a significant predictor of lower (better) component 1 scores (CAP score: $\upbeta$ = −0.8, *t* = −2.9, *p* = 0.006). Male sex was significantly associated with higher (worse) component 1 scores ($\upbeta$ = 0.9, *t* = 2.9, *p* = 0.007). UHDRS-TMS and SDMT were not significant predictors of component 1 (UHDRS-TMS: $\upbeta$ = 0.4, *t* = 1.2, *p* = 0.2; SDMT: $\upbeta$ = −0.2, *t* = −0.7, *p* = 0.48).

**Fig. 5:**
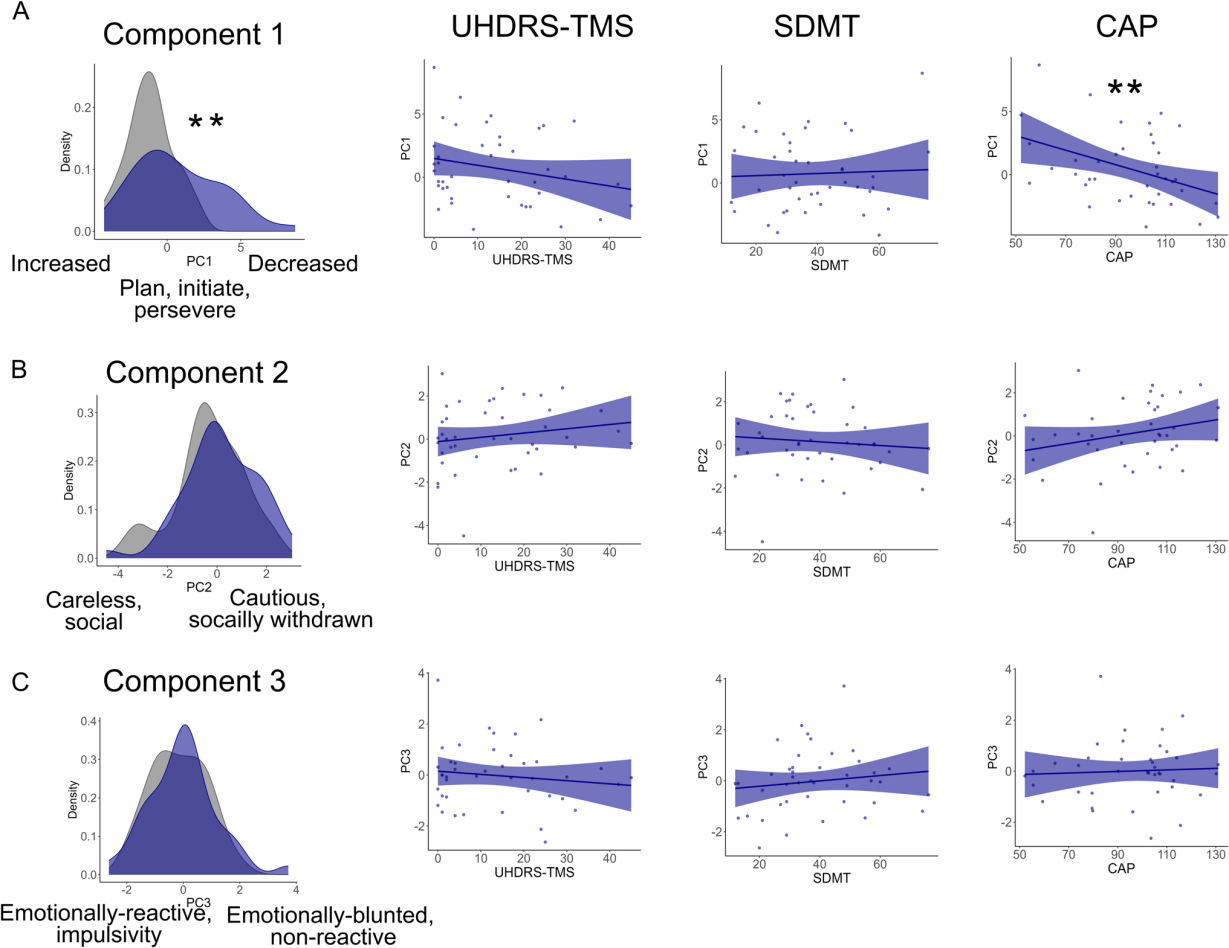
Component score associations in Huntington’s disease. Linear mixed models were fitted to component scores in the HD group only (no controls), to examine associations with disease-related variables (motor disease severity (UHDRS-TMS), cognition (SDMT), CAP score, age and sex (age and sex not shown)). A) Goal directedness deficit was significantly associated with lower CAP scores in HD (*p* = 0.006) and male sex (not shown), but not motor disease severity or cognition; B) No disease variables predicted scores on component 2; C) No disease variables predicted scores on component 3, whilst male sex (not shown) was associated with higher scores (increased emotional blunting, non-reactive). To note, differences between controls and HD were evident on component 1 only. **p < 0.01

No variables were significantly associated with component 2 scores. Male sex had significantly higher component 3 scores (emotionally blunted, nonreactive) ($\upbeta$ = 0.8, *t* = 2.4, *p* = 0.02), but no disease variables were significantly associated (Fig. 5).

### Other behaviours in HD and component scores

Given the potential overlap in HD between aspects of impulsive behaviour, perseverations and irritability, we performed exploratory analyses to examine these relationships. Linear models were fitted to component scores, with predictor variables sPBA-irritability scores and sPBA-perseveration scores, controlling for CAP score and sex. Irritability, but not perseverations was significantly associated with component 1 scores (sPBA irritability: $\upbeta$ = 0.6, *t* = 3.3, *p* = 0.002; sPBA perseverations: $\upbeta$ = 0.1, *t* = 0.4, *p* = 0.7). No significant associations between irritability or perseverations and components 2 and 3 were evident.

### Goal-directedness not associated with change in motor disease severity in HD

**Fig. 6:**
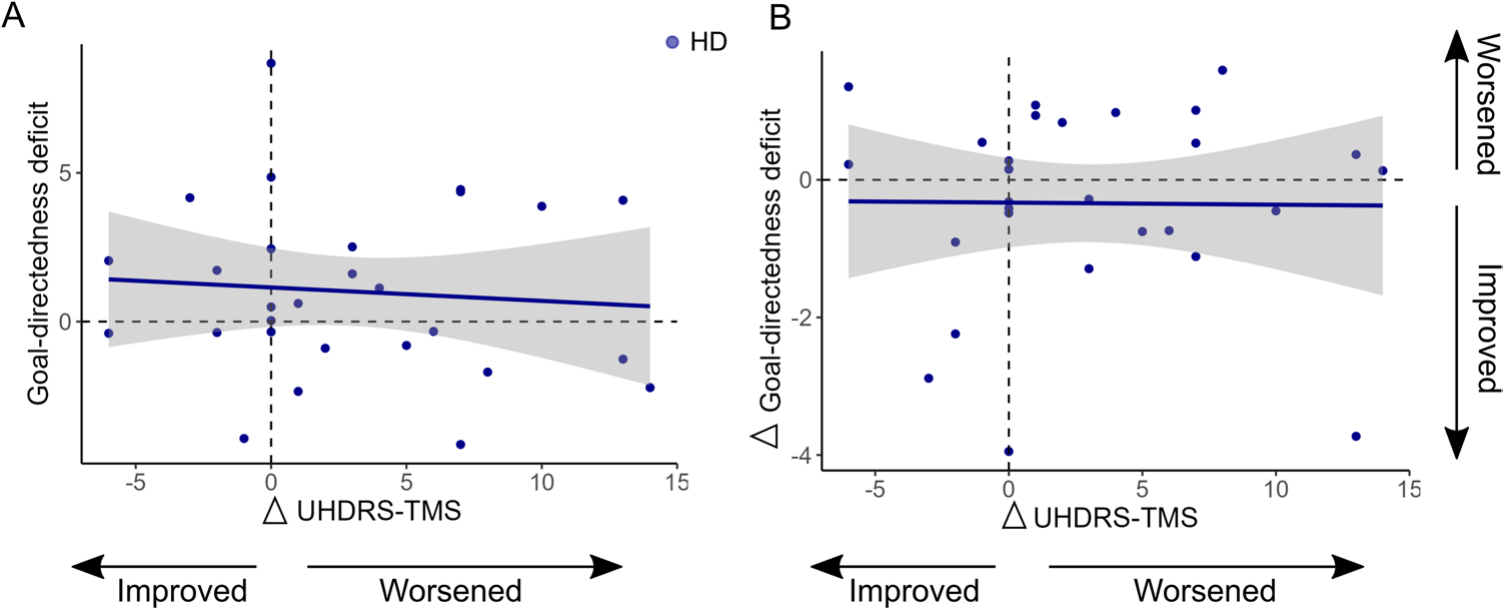
Goal-directedness and motor disease severity in Huntington’s disease **A**) Baseline goal-directedness (component 1 scores) were not associated with change in motor disease scores (UHDRS-TMS) over 1 year. **B**) Change in goal-directedness (component 1 scores) over 1 year was not associated with change in motor disease scores

Lastly, we examined whether baseline goal-directedness (component 1 scores) in HD were associated with change in motor disease severity (UHDRS-TMS) over 1 year, as well as whether individual change in goal-directedness over 1 year was associated with individual change in motor disease. No significant associations were evident (Fig. 6A & B). There was a significant worsening on average, of motor disease severity over 1 year in people with HD ($\upbeta$ = 0.23, *t* = 2.8, *p* = 0.009).

## Discussion

In the present work we examined the relationships between subscales of apathy and impulsive behaviour in people with Huntington’s disease and healthy controls. We found distinct patterns of subscale clustering when examining factor loadings from principal components analysis, that transcended the apathy and impulsive behaviour constructs. The first component described goal-directedness, contributed to by both apathy and impulsive behaviour factors, with higher scores comprising deficits in attention, planning, behavioural initiation, and perseverance. A second component described a cautious, socially withdrawn phenotype, whereas a third component described an emotional-reactive impulsive phenotype. In contrast to the first component, apathy and impulsive behaviour dimensions contributed in opposite directions to these. Linear mixed modelling revealed significantly increased scores in people with HD compared to controls on only component 1—goal-directedness—with no group differences evident on scores for components 2 and 3. This suggests that the increased apathy and impulsive behaviour that has been shown to co-occur in people with HD relates to a shared dimension of goal-directedness rather than other dimensions probed by questionnaires. All component scores did not significantly change over the 1-year period in our cohort of premanifest - mild motor manifest disease. Overall, we show that the co-occurrence of apathy and impulsive behaviour in HD relates to questionnaire measures of goal-directedness disruption, comprising attentional impulsivity, non-planning impulsivity, decreased behavioural initiation, and lack of perseverance.

In line with our hypothesis, people with HD demonstrated a goal-directedness deficit. Attention and planning (traditionally ascribed to executive functions), behavioural initiation, and perseverance are key cognitive processes underpinning the successful pursuit and attainment of goals with disruption giving rise to apathetic *and* impulsive behaviours. This component remained stable on average in HD over a 1-year period. Given the previous literature showing worsening of apathy over time in HD (Ko et al., 2023; Ruiz-Idiago et al., 2023; Tabrizi et al., 2013; van Duijn et al., 2014), we expected a goal-directedness deficit to worsen in the HD group. One reason for component stability could be the relatively mild disease stage (mean CAP score = 95) in our cohort. Despite a worsening on average of motor disease severity over 1 year, the effect size of this was small (*d* = 0.2). Particularly at this stage of HD a 1 year follow-up may not be sufficient time to capture changes in this component at a group level.

Higher (worse) component 1 scores in HD were significantly associated with lower CAP scores. Although component 1 is not apathy alone, but the shared dimensions of apathy and impulsive behaviour that loaded onto a common factor, indicating a broader deficit in goal-directedness, we nonetheless expected component 1 to worsen with disease progression. One possibility for this finding may be that our sample contains distinct phenotypes of HD at different stages—a premanifest behavioural phenotype, and a manifest motor/cognitive phenotype. Indeed, behavioural changes such as apathy and irritability are prevalent premotor manifest disease (Martinez-Horta et al., 2016), and in our sample, component 1 was associated with irritability. Another interpretation could be that the aspects of apathy that component 1 taps into—goal-directedness—are not the aspects of apathy that worsen with disease progression in HD. This finding may also in part be explained by the spectrum of milder disease in the sample.

Both cognitive and behavioural apathy loaded onto the same component in the present work, suggesting that these are similar or overlapping, rather than distinct components of apathy. Indeed, there is a paucity of evidence to suggest that cognitive and behavioural apathy are distinct dimensions of apathy (Dickson & Husain, 2022) and they are grouped together as Behaviour & Cognition in Criterion B of the revised apathy diagnostic criteria (Robert et al., 2018), which includes features pertaining to behavioural initiation and persistence.

Components 2 and 3 in the present study did not significantly differ between people with HD and controls, suggesting that whilst these components capture unique aspects of behaviour, they are not disrupted in the setting of premanifest/early HD pathology.

Whilst previously only the UPPS-P has been associated with the five-factor model of personality (Cyders & Smith, 2008), the components derived in the present study were remarkably well-aligned with three of these factors (Table 3). Component 1, which captured goal-directedness, resembled conscientiousness, component 2 resembled extraversion and component 3, neuroticism. Whilst the study of goal-directedness (in particular) dates to early philosophers, nomenclature has differed over the years as well as between fields. In the field of neuroscience, *trait* apathy and impulsivity have been examined in healthy people as a measure of their goal-directedness or motivation (Ang et al., 2017; Petitet et al., 2021), whilst neuroscientific studies of apathy and impulsivity in clinical populations have utilised apathy and impulsivity scales with clinical cut-offs to denote disrupted motivation. In contrast, in the field of personality psychology, goal-directedness/motivation has been measured as part of a broader personality assessment, under the factor “conscientiousness.”

**Table 3:** Components from the present study and the five factor model

Current Study	Five factor model (McCrae & Costa, 1985)
Component 1 *Attention* *planning* *Initiate behaviours* *Persevere towards goals*	Conscientiousness *Well-organised* *Constraint* *Will to achieve* *Impulse control*
Component 2 *Cautious, socially withdrawn* - *careless, adventure seeking, social*	Extraversion *Sociability* *Impulsivity* *Positive emotionality/excitement-seeking* *High activity level*
Component 3 *Emotionally reactive impulsivity* – *emotionally blunted and non-reactive*	Neuroticism *Experience negative emotion and thoughts* *Emotional instability*

The components from the present study were remarkably similar to three of the factors from the five factor model of personality. Component 1 resembled conscientiousness, component 2 resembled extraversion, and component 3, neuroticism.

Our second component aligned well with the personality factor of *extraversion* (McCrae & Costa, 1985). Extraversion traits range along an axis, with fun-loving, sociable, excitement-seeking on one end, and sober-minded, socially reticent, cautious on the other. Similar to the present findings, previous work examining the relationship between apathy and impulsive behaviour in the general population using raw subscale scores found that social apathy was negatively correlated with impulsivity (Petitet et al., 2021). This again points to a *cautious, socially withdrawn* phenotype. Taken together, it is clear that co-occurring apathy and impulsivity is not driven by this component, but implicated dimensions do occur at opposite ends of a behavioural spectrum.

The third component aligned with the five factor model’s *neuroticism.* Neuroticism traits include increased negative emotions and thoughts and emotional instability. Interestingly, neuroticism (including negative urgency) is a risk factor for a range of psychopathologies (Berg et al., 2015; Dudfield et al., 2023). Whether the opposite end of the spectrum would be an emotional blunting, as suggested by our third component, remains to be investigated. Like component 2, co-occurring apathy and impulsive behaviour was not captured by this component and there were no significant differences between people with HD and controls, suggesting that this component is not disrupted in the setting of (early) HD pathology. It is surprising that people with HD did not show evidence for increased emotional blunting or social withdrawal compared to controls, given that these features have previously been demonstrated in HD (Bora et al., 2016; Johnson et al., 2007). This might in part be related to the relatively mild spectrum of disease in the current study. Future work is needed to examine components two and three in a larger HD sample across stages of the disease

Studies probing the biological mechanisms underlying the five factor model show little evidence of association between these personality traits and brain structure or function (Avinun et al., 2020; Chen & Canli, 2022). Conversely, neuroscience studies have reasonably consistently identified neural substrates associated with apathy and impulsivity in clinical populations (Dalley & Robbins, 2017; Le Heron et al., 2018). Whilst personality psychology and clinical neuroscience may not share nomenclature, these fields may in fact be examining overlapping constructs, albeit at different positions along the spectrum of their expression. The biological underpinnings—of goal-directedness at least—may be meaningfully examined at the severe end of the spectrum, in the setting of neurological disease.

A limitation of the current work is that follow-up data was available at 1 year only. Further longitudinal follow-up would greatly enhance our understanding of the relationships between components identified in this study and HD. A strength of this work is the use of multiple apathy and impulsive behaviour subscales, enabling assessment of the various dimensions of these constructs, as well as the robust statistical method employed to identify co-occurrence of dimensions – principal component analysis. An important future step will be to identify the neural underpinnings of each component, particularly goal-directedness, given that it may represent the heart of co-occurring apathy and impulsive behaviour in Huntington’s disease.

Whilst apathy is generally considered a clinical construct, impulsive behaviour is not per se and can be a personality trait. Thus, these two constructs may not be directly comparable to one another. However, their common co-occurrence across a range of disorders necessitates a closer examination of which dimensions drive this co-occurrence, as in the present work. Future work should examine goal-directedness aspects of impulsive behaviour (planning, attention) in more detail and how task-derived metrics of these relate to apathy.

It is important to note that in the context of HD, apathy and impulsive behaviours may occur amidst other cognitive and psychiatric impairments. Impaired set-shifting and attention particularly, are prevalent in HD as are depression, anxiety, and irritability (McAllister et al., 2021; Paoli et al., 2017). The relationship between apathy and cognitive impairment in HD is not straightforward, however, with apathy in premanifest HD associated with cognitive decline over 24 months, but motor disease, not apathy, predictive of cognitive decline in early diagnosed HD (Andrews et al., 2021). Although we did not find significant associations between goal-directedness and cognition in the present study, one caveat is that we used a single measure (SDMT) that examines only particular aspects of cognition.

Establishing the factors that drive the co-occurrence of apathy and impulsive behaviour is an important step towards a mechanistic understanding of these common behavioural pathologies. This study demonstrates that their overlap in HD relates to a shared deficit in goal-directedness, whereas other dimensions underpinning these constructs showed opposing contributions from apathy and impulsive behaviour. Notably it was deficits in the shared goal-directedness construct—but not the others—that differed between people with HD and controls. Overall, this suggests that whilst goal-directed dimensions of apathy and impulsive behaviour co-occur, other aspects of these behaviours are evident at opposite ends of a behavioural spectrum.

## Data Availability

De-identified data is available from the corresponding author upon reasonable request. None of the experiments were preregistered.

## Footnotes

1 Perhaps an example of the *jingle fallacy* (two phenomena bearing the same name are assumed to be the same).

## Code availability

Code is available from the corresponding author upon reasonable request.
